# Explainable machine learning for precise fatigue crack tip detection

**DOI:** 10.1038/s41598-022-13275-1

**Published:** 2022-06-09

**Authors:** David Melching, Tobias Strohmann, Guillermo Requena, Eric Breitbarth

**Affiliations:** 1 German Aerospace Center (DLR), Institute of Materials Research, Linder Hoehe, 51147 Cologne, Germany; 2 Metallic Structures and Materials Systems for Aerospace Engineering, RWTH Aachen University, 52062 Aachen, Germany

**Keywords:** Mechanical engineering, Theory and computation

## Abstract

Data-driven models based on deep learning have led to tremendous breakthroughs in classical computer vision tasks and have recently made their way into natural sciences. However, the absence of domain knowledge in their inherent design significantly hinders the understanding and acceptance of these models. Nevertheless, explainability is crucial to justify the use of deep learning tools in safety-relevant applications such as aircraft component design, service and inspection. In this work, we train convolutional neural networks for crack tip detection in fatigue crack growth experiments using full-field displacement data obtained by digital image correlation. For this, we introduce the novel architecture *ParallelNets*—a network which combines segmentation and regression of the crack tip coordinates—and compare it with a classical U-Net-based architecture. Aiming for explainability, we use the Grad-CAM interpretability method to visualize the neural attention of several models. Attention heatmaps show that *ParallelNets* is able to focus on physically relevant areas like the crack tip field, which explains its superior performance in terms of accuracy, robustness, and stability.

## Introduction

Quantifying fatigue crack growth is of significant importance for evaluating the service life and damage tolerance of critical engineering structures and components that are subjected to non-constant service loads^1^. Fatigue crack propagation (*fcp*) data are usually derived from standard experiments under pure Mode I loadings. Therefore, a straight crack path is usually assumed, which can be monitored by experimental techniques such as the direct current potential drop method^2,3^. Effects like crack kinking, branching, deflection or asymmetrically growing cracks cannot be captured without further assumptions, hindering the application of classical methods for multiaxial loading conditions. Alternative methods able to capture the evolution of cracks under complex loading conditions are therefore needed.

In recent years, digital image correlation (DIC) has become instrumental for the generation of full field surface displacements and strains during *fcp* experiments^4^. Coupled to suitable material models, the DIC data can help to determine fracture mechanics parameters like stress intensity factors (SIFs)^5^, J-integral^6^ as well as local damage mechanisms around the crack tip and within the plastic zone^7,8^. All this requires a clear knowledge of the crack path and, especially, the crack tip position. Gradient-based algorithms like the Sobel edge-finding routine can be applied to identify the crack path^9^. Moreover, the characteristic strain field ahead of the crack tip can help to find the actual crack tip coordinates by fitting a truncated Williams series to the experimental data^10^. However, the precise and reliable detection of crack tips from DIC displacement data is still a challenging task due to inherent noise and artefacts in the DIC data^11^.

Convolutional neural networks (CNNs) led to enormous breakthroughs in computer vision tasks like image classification^12^, object detection^13^, or semantic segmentation^14^. Recently, deep learning algorithms are also finding their way into materials science^15^, mechanics^16,17^, physics^18^ and even fatigue crack detection: Rezaie et al.^19^ segmented crack paths directly from DIC grayscale images whereas Strohmann et al.^20^ used the physical displacement field calculated by DIC as input data to segment fatigue crack paths and crack tips. Both architectures were based on the U-Net encoder-decoder model^21^. Pierson et al.^22^ developed a CNN-based method to predict 3D crack surfaces based on microstructural and micromechanical features. Moreover, CNNs are able to segment crack features from synchrotron-tomography scans^23,24^ and can also detect fatigue cracks in steel box grinders of bridges^25^. For a detailed review on fatigue modeling and prediction using neural networks we refer to the recent review article by Chen et al.^26^.

CNNs are extremely flexible and consist of millions of tunable parameters enabling them to learn complex patterns and features. On the other hand, their depth and complexity make it very hard to explain the function representation of these models. Nevertheless, explainability and interpretability^27^ of such black-box-models are crucial to ensure their robustness and reliability as well as to detect training data biases^28^. Furthermore, it helps stakeholders gain trust in data-driven models and thus contributes to a certified and secure application of these models in the production environment.

There are several methods to approach interpretability of deep neural networks^29,30^. Gradient-weighted Class Activation Mapping (*Grad-CAM*)^31^ is one of many state-of-the-art interpretability techniques which produce visual explanations of the decisions made by CNN-based models, see, e.g., Alber et al.^32^ for a variety of other approaches. It helps users to gain trust and experts to discern stronger models from weaker ones even in case of seemingly indistinguishable predictions. The method generalizes Class Activation Mappings^33^ and was recently extended to semantic segmentation^34^, resulting, e.g., in the successful interpretation of CNN-based brain tumor segmentation models^35,36^.

In the present work, we investigate the interpretability of machine-learned fatigue crack tip detection models. For this, we introduce a novel network architecture called *ParallelNets.* The architecture is an extension of the classical segmentation network *U-Net* by Ronneberger et al.^21^ and its modification by Strohmann et al.^20^ for fatigue crack segmentation in DIC data. To this purpose, we train a parallel network for the regression and segmentation of crack tip coordinates in two-dimensional displacement field data obtained by DIC during a *fcp* experiment. Exemplarily, we use the Grad-CAM method to obtain neural attention heatmaps for input samples from several *fcp* experiments. Finally, we discuss the overall attention and the individual layer-wise attention of three trained models and find relations to their performance and robustness on unseen data.

## Methodology

### Material and data generation

The experimental data used in this work was generated during *fcp* experiments with MT-specimens of the aluminum alloy AA2024-T3. The alloy is commonly employed for aircraft fuselage structures^37^. Displacement fields were measured on the surface of the specimens during the experiments by means of a commercial 3D DIC system. Further details on the experimental conditions and the resulting DIC data can be found in Strohmann et al.^20^ and Breitbarth et al.^38^.

We use DIC displacement data from three different *fcp* experiments denoted by ${S}_{w, t}$ where $w$ is the width and $t$ the thickness of the specimen $S$ in millimeters:

- ${S}_{160, 4.7}$ (Strohmann et al.^20^).
- ${S}_{160, 2.0}$ (Strohmann et al.^20^).
- ${S}_{\mathrm{950,1.6}}$ (Breitbarth et al.^38^).

For the first two experiments (${S}_{160, 4.7}$, ${S}_{160, 2.0}$) the image acquisition rate was controlled by the crack length. The crack length was determined by the direct current potential drop method using Johnson’s equation^39^ A series of 5 images was acquired every 0.2 mm of crack extension starting at maximal force followed by four successive load steps (75%, 50%, 25%, and 10%). We refer to Strohmann et al.^20^ for further details on the experimental setup and data generation for these two experiments.

The specimen size in the third experiment (${S}_{\mathrm{950,1.6}}$) differs considerably from the first two (950 mm in comparison to 160 mm width). The large specimen was used to investigate very high SIFs (up to $\sim$ 130 MPa√m) at load ratios $R$ = 0.1, 0.3, and 0.5. In the present work, we use the experimental data from the load ratio $R$ = 0.3.

### Ground truth

Ground truth data for the crack tip position was obtained by manual segmentation of high-resolution optical images^20^. Here, we use the ground truth data from experiment ${S}_{160, 4.7}$ for training and validation (i.e. model selection).

Since the segmentation of one crack tip located in one pixel within an array of 256 $\times$ 256 pixels (size of the interpolated displacement field acquired by DIC) suffers from severe class imbalance^40^ ($\sim$ 1:50 k), we artificially increased the number of crack tip pixels by labeling a surrounding 3 $\times$ 3 pixel grid as class “crack tip” resulting in an imbalance of $\sim$ 1:7300. This imbalance is handled by using the Dice loss function (see “Loss”). Such a 3 × 3 grid is also necessary for data augmentation purposes, especially random rotation, since single pixels might otherwise get lost during rotation and interpolation.

### Network architecture

There are at least two different approaches to design a neural network for the prediction of crack tips in displacement field data:

1. We can view this task as a regression problem and combine a convolutional neural feature extractor with a fully connected regressor that outputs the crack tip position^41^. Such architectures were already used for image orientation estimation^42^, pose estimation^43^ or, more recently, respiratory pathology detection^44^. This approach can be advantageous since it overcomes the class imbalance problem. However, we found that such models are not precise enough for our use case and they are useless for images without crack tips or with multiple cracks.
2. We can use a semantic segmentation network like in Strohmann et al.^20^ to segment pixels of class “crack tip”. This approach has advantages when it comes to precision. However, the high class imbalance in our data makes the training of the network difficult.

#### ParallelNets

**Figure 1:**
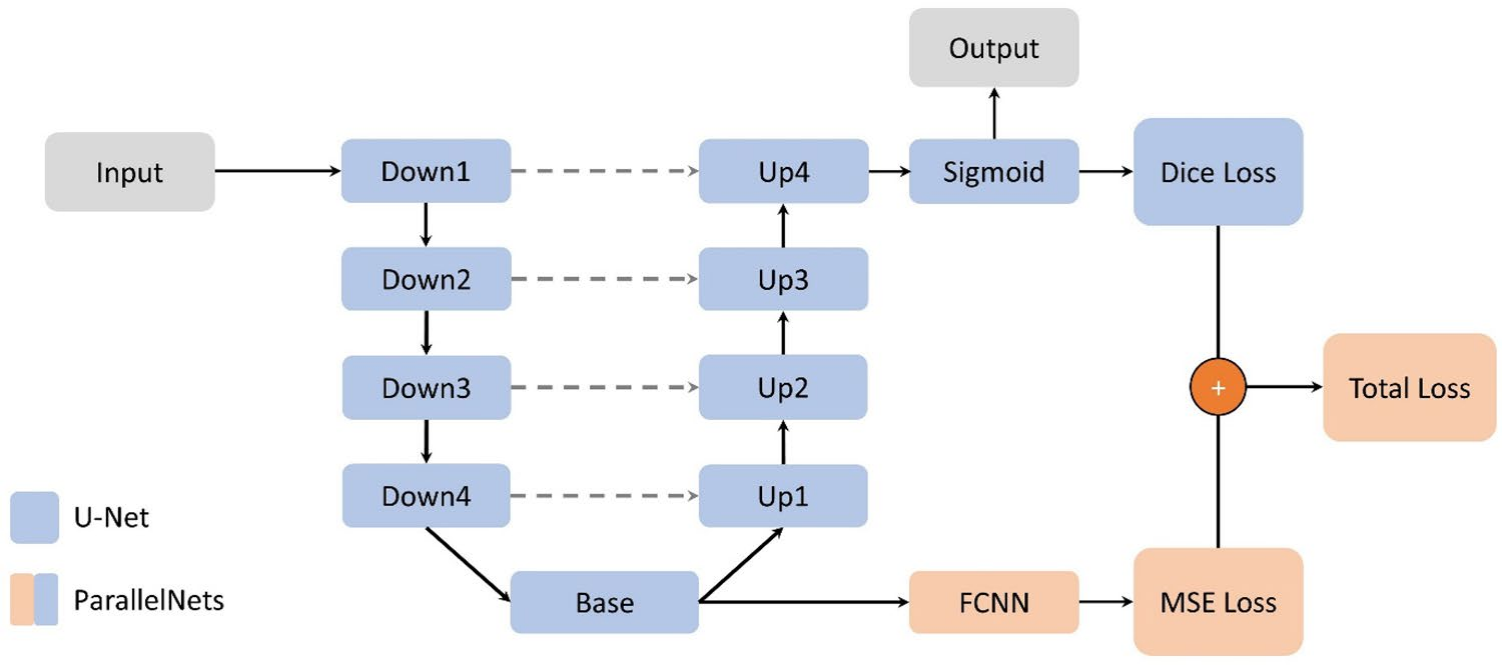
Schematic ParallelNets architecture. The classical U-Net architecture^21^ with four encoder blocks (Down) and four decoder blocks (Up) connected by a base block (Base) is shown in blue. Encoder and decoder blocks of the same level are connected by skip connections (gray dashed lines). The additional modules of our ParallelNets architecture are shown in orange and basically consist of a fully connected neural network (FCNN) which is trained to output the crack tip position in terms of normalized x and y coordinates.

We introduce an architecture named *ParallelNets* that combines the two approaches described above and train them in a parallel network^45,46^*.* The architecture is shown in Fig. 1: a classical U-Net^21^ encoder-decoder model is fused with a Fully Connected Neural Network (FCNN) based at the bottleneck of the U-Net. Consequently, the network has two output blocks, i.e. a crack tip *segmentation* from the U-Net decoder and a crack tip *position* from the FCNN regressor. On the one hand, we expect that this *learning redundancy* can lead to improved robustness because the network encoder needs to provide good latent representations for both tasks, namely segmentation and regression. On the other hand, for the same reason *ParallelNets* might be harder to train than a simple U-Net and the corresponding segmentation and regression losses need to be properly balanced.

The U-Net consists of four encoder blocks *Down1, …, Down4* and corresponding decoder blocks *Up1, …, Up4*. They are joined by a *Base* which consists of two consecutive CNN blocks between which we use dropout^47^. Encoder and decoder blocks of matching resolution are connected via skip connections to allow an efficient flow of information through the network. These connections increase segmentation quality^48^. Following Strohmann et al.^20^, we use LeakyReLU instead of the original ReLU as activation function for our U-Net architecture.

The FCNN consists of an adaptive average pooling layer followed by two fully connected layers with ReLU activation functions and finishing with a 2-neuron linear output layer. It predicts the (normalized) crack tip position $y=\left({y}_{1},{y}_{2}\right) \in {[-\mathrm{1,1}]}^{2}$ relative to the center of the input data.

#### Loss

During training, we calculate the mean squared error between the prediction and the ground truth crack tip position $\widehat{y}=\left({\widehat{y}}_{1},{\widehat{y}}_{2}\right)\in {[-\mathrm{1,1}]}^{2}$, i.e.

1 $$\mathrm{MSE}\left(y, \widehat{y}\right)= \sqrt{{({y}_{1}-{\widehat{y}}_{1})}^{2}+{({y}_{2}-{\widehat{y}}_{2})}^{2}}$$

Since the segmentation problem is highly imbalanced, we use Dice loss^49^ for the segmentation output:

2 $$\mathrm{Dice}\left(z,\widehat{z}\right)=1-\frac{2 {\sum }_{ij}{z}_{ij}{\widehat{z}}_{ij}+\varepsilon }{{\sum }_{ij}{(z}_{ij}+{\widehat{z}}_{ij})+\varepsilon }$$

where $z=\left({z}_{ij}\right)$ with ${z}_{ij}\in [\mathrm{0,1}]$ denotes the segmentation output (after sigmoid activation) and $\widehat{z}=\left({\widehat{z}}_{ij}\right)$ stands for the ground truth. Here, $\varepsilon >0$ is a small constant introduced to treat the edge case $z=\widehat{z}\equiv 0$. We chose $\varepsilon ={10}^{-6}$. These two losses are then combined into a (weighted) total loss

3 $${\mathrm{Loss}}_{\omega }(z,y,\widehat{z},\widehat{y})=\mathrm{ Dice}\left(z,\widehat{z}\right)+\omega \mathrm{MSE}\left(y, \widehat{y}\right)$$

where $\omega \ge 0$ is a weight factor which tunes the training influence of the FCNN. If $\omega =0$, the parallel FCNN branch is inactive and the *ParallelNets* is reduced to the classical U-Net.

### Data augmentation and normalization

First, each input displacement fields ${u}_{x}$ and ${u}_{y}$ are interpolated on a regular 256 $\times$ 256 grid. We perform a data normalization in combination with the following consecutive data augmentation steps of the DIC dataset:

1. **Random crop** of the input with a crop size between 120 and 180 pixels where the left edge is chosen randomly between 10 to 30 pixels.
2. **Random rotation** by an angle between − 10 and 10 degrees and subsequently crop the largest possible square from the rotated input.
3. **Random flip** up/down with a probability of 50%.

Since random crop and random rotation reduce the input size, we need to up-sample the input and ground truth by means of a linear and nearest neighbor interpolation to a multiple of 16. We choose 224 $\times$ 224. A further up-sampling to the original size of 256 $\times$ 256 would only result in more interpolated data points. Moreover, a reduced input size yields less GPU memory, and thus speeds up training.

No data augmentation is used during validation and the input data stays at their original size.

### Datasets and data splitting

The data generated during the *fcp* experiments introduced in “Material and data generation” was split into the following four datasets (the term *sample* indicates hereafter individual DIC images acquired at a single load condition):

1. Training dataset ${train}_{\mathrm{160,4.7},\mathrm{right}}$: The data acquired from the right side of the specimen ${S}_{\mathrm{160,4.7}}$ consisting of 835 labeled samples.
2. Validation dataset ${val}_{\mathrm{160,4.7},\mathrm{left}}$: The data acquired from the left side of the specimen ${S}_{\mathrm{160,4.7}}$, also consisting of 835 labeled samples.
3. Test dataset ${test}_{\mathrm{160,2.0}}$: Data acquired from the left and right sides of the specimen ${S}_{\mathrm{160,2.0}}$ with 2 $\times$ 1410 = 2820 samples.
4. Test dataset ${test}_{\mathrm{950,1.6}}$: Data acquired from the left and right sides of the specimen ${S}_{\mathrm{950,1.6}}$ with 2 $\times$ 204 = 408 samples.

The data of the left side of the specimens are preprocessed to guarantee a data distribution similar to the right side. Both displacement fields ${u}_{x}$ and ${u}_{y}$ are mirrored along the $y$-axis and the $x$-displacements are multiplied by − 1.

### Architecture optimization and training

After manual architecture optimization of the number of initial feature channels and the number of hidden layers and neurons of the FCNN, we selected 64 initial feature channels for the U-Net and 2 hidden layers for the FCNN consisting of 1024 and 256 neurons, respectively.

To train *ParallelNets* properly, we found by trial-and-error that a loss weight of $\omega =100$ works well, since it balances both loss terms making the whole model learn both the segmentation and regression of crack tips. Lower values of $\omega$ pronounce the segmentation task and higher values the regression task.

In terms of hyperparameter optimization, we identified the Adam optimizer^50^ with a learning rate of $5\times {10}^{-4}$ and a batch size of 16 by trial-and-error. Moreover, we tried different dropout probabilities $p\in [0,\frac{1}{2}]$ for the bottleneck of U-Net and *ParallelNets* but found no substantial difference.

We trained several randomly initialized U-Nets and *ParallelNets* for 500 epochs on the dataset ${train}_{\mathrm{160,4.7},\mathrm{right}}$. After each epoch, the networks were evaluated on ${val}_{\mathrm{160,4.7},\mathrm{left}}$ and finally the network with the smallest validation Dice loss was selected.

### Grad-CAM method

**Figure 2:**
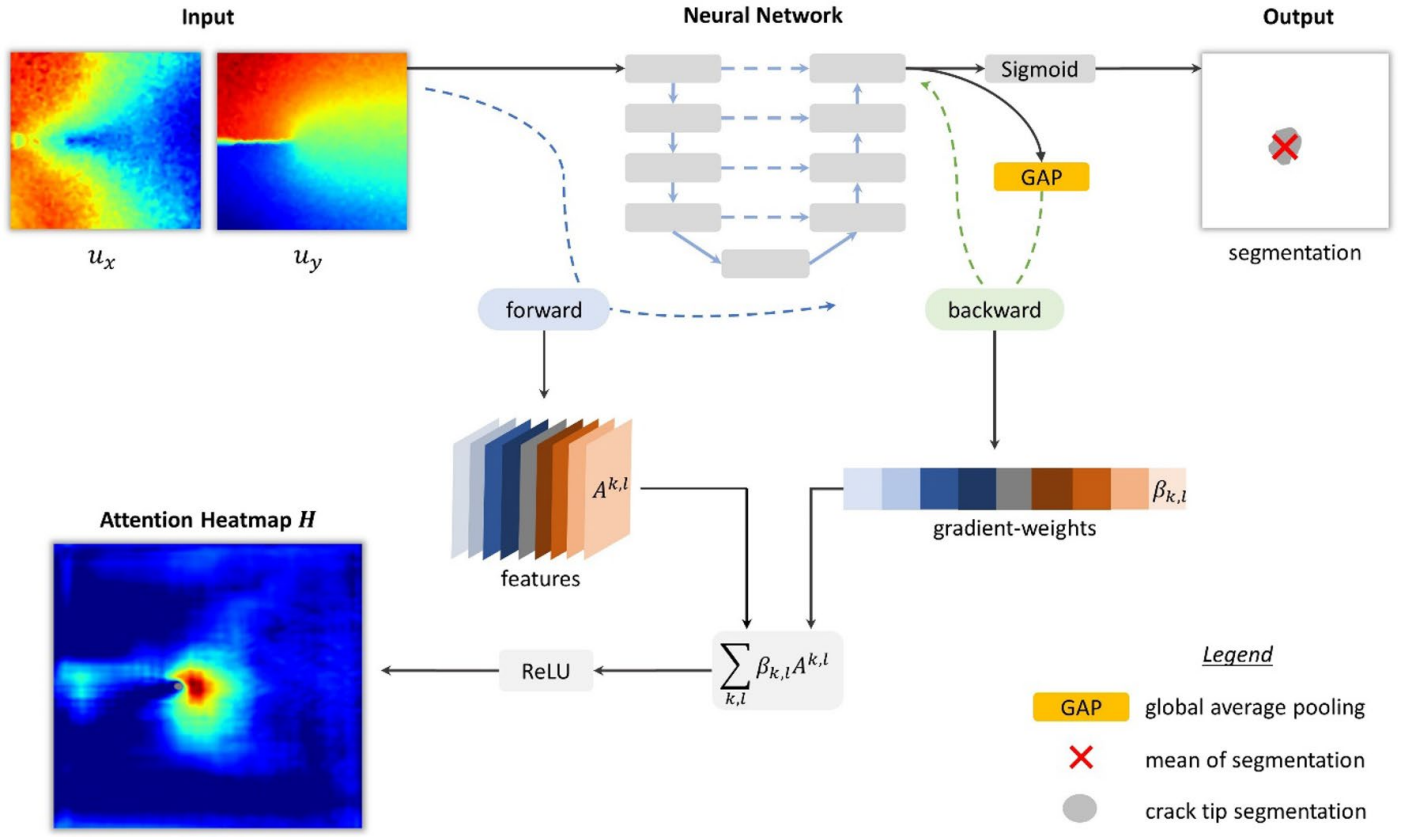
Grad-CAM method for visualization of deep neural network’s attention. Internal features of the neural network collected during a forward pass of input data are combined by weighting with average pooled gradients computed during a backward pass.

We use the so-called *Grad-CAM*^31^ method to interpret the results. This method allows quantification and visualization of the spatial attention of deep neural networks used for segmentation tasks. Classically, the algorithm is used to produce layer-wise attention heatmaps^35,36^. Figure 2 shows the workflow of the network and the Grad-CAM method.

To obtain the attention heatmap $H\left(u\right)$ for input displacements $u=({u}_{x},{u}_{y})$, we first collect the internal features from selected layers during the forward pass. The network output $\Phi (u)$ (before Sigmoid activation) is then global average-pooled (GAP) over the size of the image to get the scalar output score

4 $$\varphi (u)=\frac{1}{N}\sum_{i,j}{\Phi }_{ij}\left(u\right).$$

where $N$ denotes the number of pixels of the output. The score is backpropagated through the network to calculate the gradients $\frac{\partial \varphi }{\partial {A}^{kl}}$ with respect to the feature activation maps ${A}^{kl}$ of the $k$-th filter and $l$-th layer. These gradients are then global average-pooled over their width and height dimensions (indexed by $i,j$) to obtain the gradient-weights

5 $${\beta }_{kl}\left(u\right)=\frac{1}{{N}_{l}}\sum_{i,j} \frac{\partial \varphi }{\partial {A}_{ij}^{kl}}\left(u\right),$$

where ${N}_{l}$ denotes the number of pixels of the features of the respective layer. These weights ${\beta }_{kl}$ capture the importance of the feature ${A}^{kl}$ for the segmentation score $\varphi$. Finally, we compute the attention map by applying the $\mathrm{ReLU}$ activation function to the gradient-weighted sum of features:

6 $$H\left(u\right)=\mathrm{ReLU}\left(\sum_{k,l}{\beta }_{kl}\left(u\right){A}^{kl}(u)\right)$$

Here, the function $\mathrm{ReLU}\left(x\right)=\mathrm{max}(x,0)$ is applied to highlight areas which have a positive influence on the output score $\varphi$.

## Results and discussion

If we fix a network architecture and train several randomly initialized models, the results in terms of final loss and accuracy are stable. However, the network attention substantially differs for each trained model. This behavior is expected^31^. In fact, these differences in terms of attention can be used to successfully discern stronger models from weaker ones even if both make almost identical predictions.

In our study, we observed three main behaviors:

i. instable crack path attention.
ii. stable crack path attention.
iii. stable crack tip field attention.

To illustrate these differences, we select three representative trained models to discuss various performance and explainability results. Two of the three models were trained with the U-Net architecture and are denoted as *U-Net-1* (dropout probability $p=0.25$) and *U-Net-2* ($p=0.5$). The third one was trained with the *ParallelNets* architecture with $p=0.2$ (see “ParallelNets”) and is referred to as *ParallelNets-1. *The latter possesses two outputs, namely the encoder-decoder segmentation and the FCNN regression of the crack tip position (Fig. 1). For simplicity, we only use the segmentation output because it turned out to be more precise than the regression output. However, it might be advantageous to use the regression output as an additional backup prediction in cases where the crack tip segmentation fails or to select the most likely crack tip region (cf. Section 2.8 of Strohmann et al.^20^). The evolution of the attention obtained by the Grad-CAM method for the three networks can be seen in the supplementary videos together with the crack tip segmentation as the fatigue crack grows. We randomly selected three representative input samples at maximal load from the different datasets for further analysis:

- ${val}_{547}$ (short $val$)—stage number 547 of the validation dataset, which corresponds to the left side of specimen ${S}_{160, 4.7}$.
- ${test}_{\mathrm{160,2.0}, \mathrm{left}, 1000}$ (short ${test}_{\mathrm{small}}$)—stage number 1000 of the left side of the small specimen ${S}_{160, 2.0}$.
- ${test}_{\mathrm{950,1.6}, \mathrm{left},290}$ (short ${test}_{\mathrm{large}}$)—stage number 290 of the left side of the larger specimen ${S}_{950, 1.6}$.

**Figure 3:**
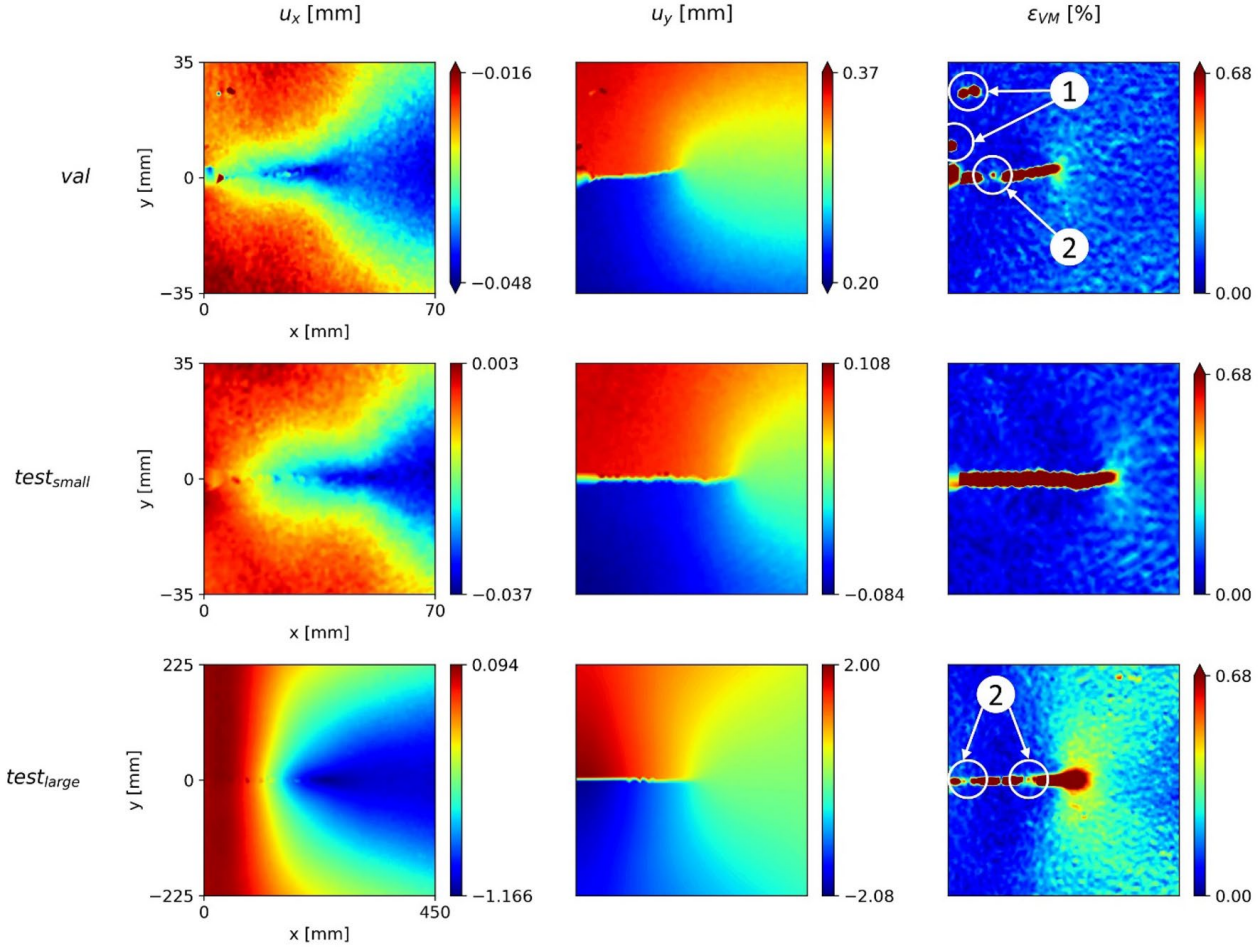
Three input data samples acquired by digital image correlation during different fcp experiments. The first and second columns show the $x$ and $y$ displacement fields and the third column the von Mises equivalent strain fields.

Figure 3 shows the displacements and von Mises equivalent strain acquired by DIC for the three samples. The results are interpolated on a 256 $\times$ 256 pixels grid. While the samples are qualitatively similar, it has to be considered that the size of the MT-specimen for ${test}_{\mathrm{large}}$ is six times larger than the others. The deformation field around the crack tip is best visible in the von Mises equivalent strain field in Fig. 3.

There are several issues in the DIC data that must be considered: first, inherent noise often hinders a correct distinction between relevant features and artefacts, particularly at low strains. For instance, the large strains (red) in the vicinity of the crack path (marked as ①) are artefacts arising from a locally flawed black and white pattern. In addition, the strain next to the crack path has no physical meaning because neighboring DIC facets are not connected, which leads to the calculation of unrealistically large strains. This red area often shows random gaps along the crack path (see the regions marked as ②). In reality, however, the crack faces are traction-free.

### Attention results

**Figure 4:**
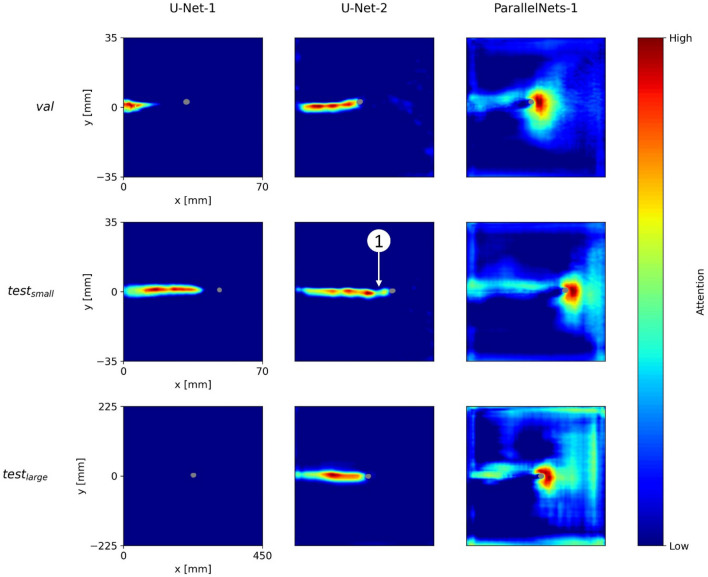
Grad-CAM attention heatmaps for the three trained networks (columns) and three different input samples (rows). The segmented crack tips are shown in gray.

Figure 4 shows the (overall) network attention heatmaps of the models *U-Net-1*, *U-Net-2*, and *ParallelNets-1* for the three input samples shown in Fig. 3. The segmented crack tip pixels are shown in gray. In contrast to layer-wise attention heatmaps^35^, these network attention heatmaps are computed with internal features from all encoder–decoder blocks of the neural networks, i.e. the output feature activations of Down1, Down2, Down3, Down4, Base, Up1, Up2, Up3, and Up4 (see Fig. 1). While all three models predict a position of the crack tip, their network attention heatmaps are distinctively different. This phenomenon was already observed in other works^28,31^.

We find that *U-Net-1* displays inconsistent attention heatmaps. On the one hand, for $val$ and ${test}_{\mathrm{small}}$ the model seems to pay attention to different parts of the crack path. On the other hand, there are no areas of high attention for ${test}_{\mathrm{large}}$. This result indicates the confusion of *U-Net-1* in the evaluation of ${test}_{\mathrm{large}}$ which may be related to the larger specimen dimensions.

Moreover, we see that *U-Net-2* consistently focuses on the crack path. The output segmentation is always located right in front of the area of high attention. Nevertheless, there are attention gaps along the crack path, e.g. the region right behind the segmented tip in ${test}_{\mathrm{small}}$ ①. Such gaps might result from DIC artefacts and correlate well with stability issues which are discussed in “Stability and robustness”.

Finally, we observe that *ParallelNets-1* focusses its attention on the area ahead and around the crack tip. This attention is consistent for all three samples and suggests that the neural network is able to identify the physical crack tip near-field^51^ in front of the crack. Such attention behavior was only found in models trained with the *ParallelNets* architecture and is desirable for the following reason: our training data is biased in the sense that each sample contains exactly one crack tip. We observed that models which focus their attention on the crack path erroneously segment a crack tip in cases where the crack path is visible but the tip is actually located outside the image. Supplementary Figure S1 shows an example of a false crack tip segmentation of *U-Net-2* in case the model’s field of view is restricted to $x\le 40$ and $-20\le y\le 20$. Here, *ParallelNets-1* correctly predicts no crack tip.

### Performance results

We choose the following metrics for evaluation of model performance on the training, validation and test datasets:

- **Dice coefficient** defined as $DSC :=(1-\mathrm{Dice})$ (see Eq. (2))
- **Reliability** of crack detection, calculated as the number of input samples with at least one pixel segmented as crack tip over the total number of input samples (every sample contains one crack tip).
  This metric is particularly interesting because it can be computed without any ground truth and determines whether the network has overfitted the training data. Moreover, it can indicate if a model undersegments, which is a common problem in imbalanced segmentation tasks.
- **Deviation** from the ground truth crack tip position in millimeters. The prediction position is calculated as the mean position of all pixels segmented as “crack tip”. More elaborate postprocessing steps which first select the most likely crack tip region^20^ are not considered here. If no pixel is segmented by the model the corresponding sample is skipped. Consequently, less reliable models may achieve smaller mean deviations over a whole dataset as the difficult samples are excluded. This effect should be considered when assessing model performance.

The results are shown in Table 1. We are only able to calculate the Dice coefficient and deviation for the training and validation datasets since the test datasets are unlabeled. *ParallelNets-1* outperforms the other networks on all datasets except the validation dataset. Especially, it is the most reliable network on unseen data (${test}_{\mathrm{160,2.0}}$ and ${test}_{\mathrm{950,1.6}}$) and reaches a perfect reliability on the training dataset. An overall test reliability of 96.8% is reached on the unseen data. Furthermore, in terms of accuracy, it shows an overall mean deviation of the crack tip position from the ground truth of 0.54 mm (training and validation data combined) with a standard deviation (*std*) of 0.38 mm. The model generalizes correctly also to larger specimen sizes ($tes{t}_{\mathrm{950,1.6}})$, although, in contrast to Strohmann et al.^20^, no additional synthetic training data in form of finite element simulations was needed.

**Table 1:**
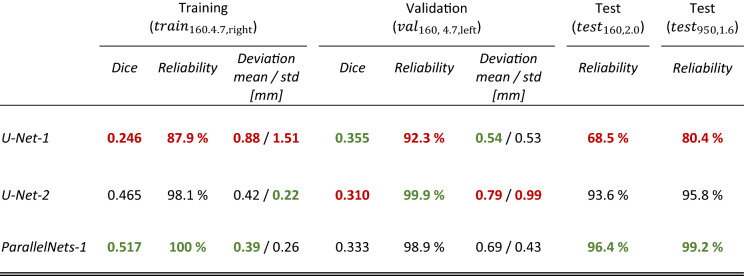
Performance comparison of the three trained models on training, validation, and test datasets with respect to the Dice coefficient (higher is better), reliability (higher is better), and mean deviation from crack tip ground truth in millimeters (lower is better).

The second-best network is *U-Net-2* with a deviation of the crack tip position (*mean*/*std*) of 0.61/0.74 mm and an overall test reliability of 93.9% on unseen data. *U-Net-1* shows the best performance only for the Dice coefficient and deviation on the validation dataset. We remark again that the networks were selected during training using the validation Dice loss as the only selection criterion. This explains why the network *U-Net-1* was chosen although it is far less reliable (70% overall test reliability on unseen data) and least accurate on the training dataset (0.88 mm mean deviation). This shows the need for improved model selection criteria during or after training.

### Stability and robustness

We now compare the crack detection stability of the different models. The detected crack tip positions should result in a growing crack length, i.e. the crack length $a$ increases between subsequent samples, i.e. $\Delta a={a}_{new}-{a}_{old}$ should be positive. We estimate the crack length

$$a\approx \sqrt{{({x}_{\mathrm{tip}}-{x}_{\mathrm{o}})}^{2}+{({y}_{\mathrm{tip}}-{y}_{\mathrm{o}})}^{2}},$$

where (${x}_{\mathrm{tip}},{y}_{\mathrm{tip}}$) and (${x}_{\mathrm{o}},{y}_{\mathrm{o}}$) denote the coordinates of the crack tip and the crack origin, respectively. We expect $\Delta a$ to be centered around 0.2 mm for the training, validation and ${test}_{\mathrm{160,2.0}}$ datasets, which was the crack growth increment between subsequent images. The length of the crack during the *fcp* experiment of the test set ${test}_{\mathrm{950,1.6}}$ was not used to control image acquisition. Therefore, this experiment is excluded from the stability study.

**Figure 5:**
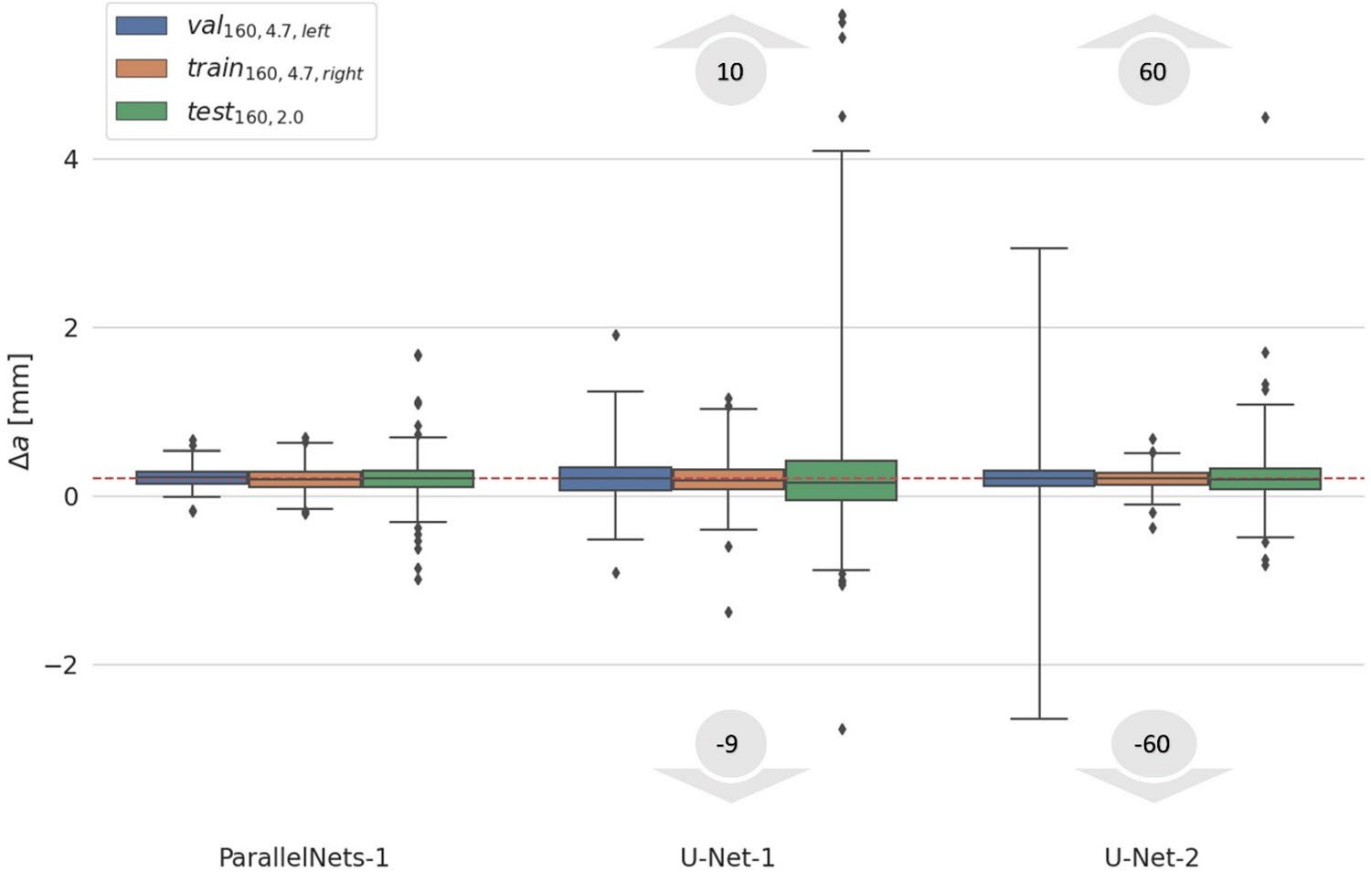
Stability of crack detection models between subsequent steps at maximal force. The target baseline for $\Delta \mathrm{a}$ is 0.2 mm depicted as a red dashed line. Quartiles (25–75%) are shown as colored boxes. The vertical black-line intervals indicate the 1–99% quantiles. Diamonds show outliers. For the models U-Net-1 and U-Net-2 these outliers actually range from − 9 to 10 and − 60 to 60 mm, respectively, and partially lie outside the plotted range.

Figure 5 shows a boxplot of $\Delta a$ for the three models and three different datasets. The results show that the mean $\Delta a$ for all distributions reflects the crack growth expectation of $\sim$ 0.2 mm. However, *ParallelNets-1* has the narrowest distribution proving its superior stability (see also standard deviation in Table 1). In contrast, the models *U-Net-1* and *U-Net-2* produce outliers which range from − 9 to 10 and − 60 to 60 mm, respectively. This behavior can be explained by the models’ attention heatmaps. Focusing on the crack path, the networks *U-Net-1* and *U-Net-2* can be confused more easily by artefacts along the crack path, which make the predictions jump back and forth between subsequent steps forming pairs of outliers (e.g. − 60 and 60 mm).

### Layer-wise network attention

So far, we have only considered overall network attention. More specifically, we collected the internal activations of all major network blocks and combined them into a single attention heatmap. This approach enhances explainability while hampering faithfulness^31^ of the visualization. In order to get a deeper insight into the networks’ actual attention mechanisms and functioning, we need to look at layer-wise attention heatmaps^35^. These layer-wise visualizations are calculated with the Grad-CAM method by restricting the features ${A}_{k,l}$ to one internal block of the network. For a better overview, we only present the three most relevant blocks for each model, i.e. the blocks for which the attention is quantitatively the highest in comparison to other blocks.

**Figure 6:**
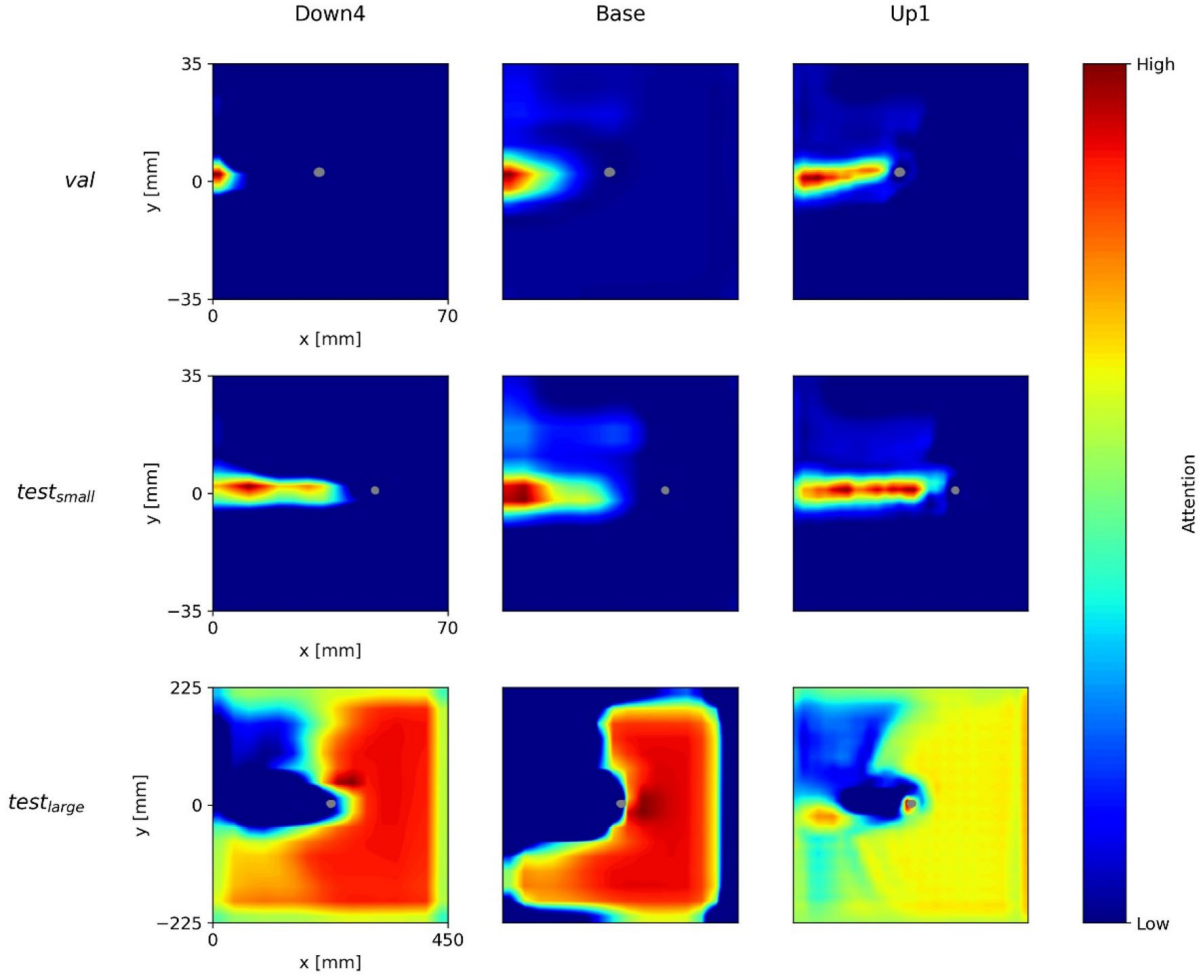
Network attention of U-Net-1’s layers Down4, Base, and Up1 for the three DIC input samples above.

Figure 6 shows the attention of *U-Net-1* for the blocks *Down4*, *Base*, and *Up1* (see Fig. 1). We see that the attention is inconsistent between the three samples. Especially the visualization of the larger MT-specimen’s sample (${test}_{\mathrm{large}}$) is very different from the other two samples ($val$, ${test}_{\mathrm{small}}$). Furthermore, for $val$ and ${test}_{\mathrm{small}}$ the attention is focused on the crack path at significant distance to the predicted crack tip segmentation.

**Figure 7:**
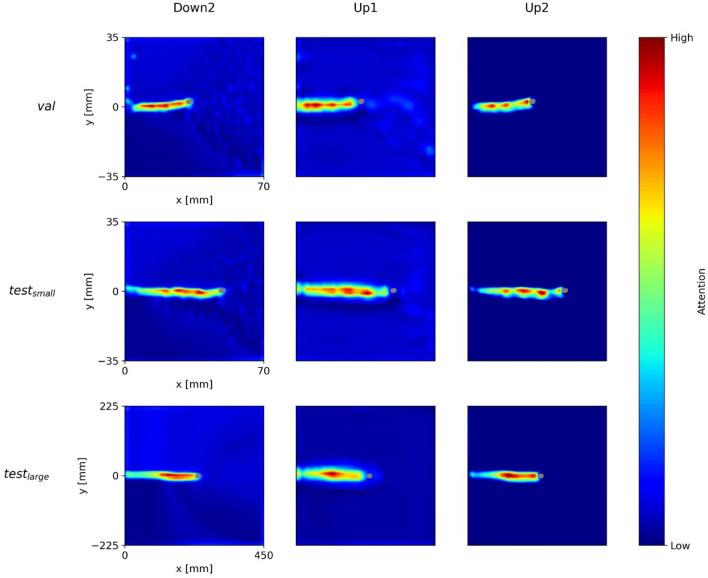
U-Net-2’s network attention of the layers Down2, Up1, and Up2 for the three DIC input samples above.

In Fig. 7, we see the layer-wise attention of *U-Net-2* for the three blocks with the highest attentions, i.e. *Down2*, *Up1*, and *Up2*. In contrast to *U-Net-1*, this model shows a consistent layer-wise attention. The model is explainable in the sense that it simply focusses on the crack path to predict the crack tip. However, this can be critical in the presence of artefacts in the DIC data around the crack faces (see Fig. 3).

**Figure 8:**
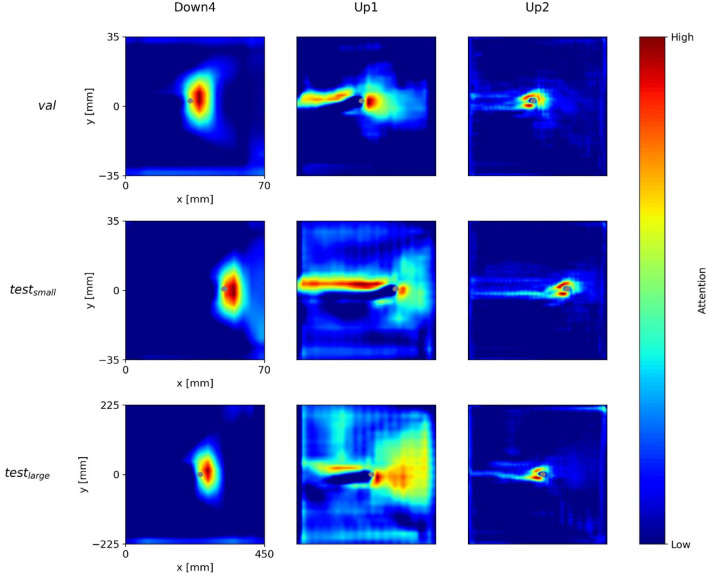
ParallelNets-1’s network attention of the layers Down4, Up1, and Up2 for the three DIC input samples above.

Figure 8 illustrates the attention of *ParallelNets-1* for the blocks—*Down4*, *Up1*, and *Up2.* The layer-wise attention shows a more versatile behavior than for the U-Net models. The block *Down4* focuses on the field ahead of the crack tip, while *Up1* pays attention to the upper part of the crack path and to a broader field in front of the crack tip. Apparently, *Up2* learned to identify the close area around the crack tip at its opening side. This feature resembles the crack tip opening displacement (CTOD) measurement technique^52^.

These findings support and explain the results shown in Fig. 4 and discussed in “Attention results”: it is evident that the network *ParallelNets-1* has learned higher order semantics. In contrast to *U-Net-2*, it displays more diverse attention on the individual layers. We conclude that this diversity leads to an increased stability and robustness of the model due to the fact that its final segmentation decision bases on several different patterns rather than merely on the detection of the crack path.

## Conclusions

We introduced the novel parallel segmentation-regression architecture *ParallelNets* and trained it to precisely detect crack tips in DIC displacement fields obtained during fatigue crack propagation experiments. We observed superior performance of this network over similarly trained classical U-Nets and searched for explanations insight the deep internal features of these models. To this purpose, we implemented two variants of the interpretability method Grad-CAM: The first one focusing on the overall network attention and the second one targeting specific blocks of the network for their interpretability.

Considering the results in “Results and discussion”, the following conclusions can be drawn:

1. **Network architecture:** For our specific application, where the problem of finding a crack tip position can be tackled either by regression or segmentation models, we find that a combination of these two strategies into a single deep end-to-end model has great benefits. In a nutshell, the parallel regression in *ParallelNets* enhances the learning of complex features thus leading to improved segmentation results.
2. **Visualization:** Grad-CAM can be used to produce meaningful and useful visualizations of neural network attention for CNN-based segmentation networks trained to segment crack tips in DIC displacements data. The algorithm can be applied to generate overall network attention heatmaps as well as layer-wise attention heatmaps.
3. **Interpretability and explainability:** These attention visualizations help human experts to identify the most promising models and can contribute to the demystification of machine-learned black-box models.
4. **Robustness and model selection:** Models focusing on physically relevant parts like the deformation field ahead and around a crack tip (*ParallelNets-1*) are more robust with respect to unseen data. The Grad-CAM method opens the possibility to identify these superior models by their attention heatmaps. This can be done during postprocessing in a machine learning pipeline or possibly even during training. Hence, we are able to produce a single network attention heatmap suitable for fast model selection and easy monitoring.

These advances pave the way towards better model selection and deeper understanding of CNN models for crack detection in safety-relevant applications and ultimately contribute to an autonomous inspection of engineering structures and components.

## Supplementary Information

Supplementary Information 1.

Supplementary Video 1.

Supplementary Video 2.

Supplementary Video 3.

Supplementary Video 4.

Supplementary Video 5.

Supplementary Video 6.

Supplementary Video 7.

Supplementary Video 8.

Supplementary Video 9.

## Data Availability

All datasets and code are publically available at 10.5281/zenodo.5740216.

## Abbreviations

$$a$$: Crack length
$\Delta a$: Crack length increment
${A}^{kl}$: Feature activation maps
$${\beta }_{kl}$$: Gradient weights
CNN: Convolutional neural network
DIC: Digital image correlation
$$\mathrm{Dice}$$: Dice loss
*DSC*: Dice coefficient
${\varepsilon }_{\mathrm{VM}}$: Von Mises equivalent strain
FCNN: Fully connected neural network
*fcp*: Fatigue crack propagation
GAP: Global average pooling
Grad-CAM: Gradient-weighted class activation mapping
$$H$$: Attention heatmap
$n,m$: Width and height of input displacements
$\mathrm{MSE}$: Mean squared error loss
MT: Middle tension
LeakyReLU: Rectified linear unit with slope 0.01
${\mathrm{Loss}}_{\omega }$: Total loss with weight factor $\omega$
$\omega$: Weight factor in combined loss
$$p$$: Dropout probability
$\varphi$: Global average-pooled network output
$\Phi$: Network output before Sigmoid activation
$$R$$: Load ratio
ReLU: Rectified linear unit
SIF: Stress intensity factor
${S}_{w, t}$: MT-specimen with width $w$ and thickness $t$ in millimeters
${test}_{w,t}$: Test dataset from specimen ${S}_{w, t}$
${test}_{\mathrm{small}}$: Test sample of the dataset ${test}_{160, 2.0}$
${test}_{\mathrm{large}}$: Test sample of the dataset ${test}_{950, 1.6}$
$${train}_{\mathrm{160,4.7},\mathrm{right}}$$: Training dataset
$$u,{u}_{x}, {u}_{y}$$: Displacements
$${val}_{\mathrm{160,4.7},\mathrm{left}}$$: Validation dataset
$val$: Test sample of validation dataset
$y, \widehat{y}$: Normalized crack tip position output and respective ground truth
$z, \widehat{z}$: Segmentation output and respective ground truth
