# Supplementary material for: Explainable machine learning for precise fatigue crack tip detection
Source: Sci Rep. 2022 Jun 9;12:9513. doi: 10.1038/s41598-022-13275-1 (PMC9184622; doi:10.1038/s41598-022-13275-1)
Supplement: Supplementary file 1 — Supplementary Information 1. [file 41598_2022_13275_MOESM1_ESM.pdf]

Explainable machine learning for precise fatigue crack tip detection: supplementary material

David Melching[1], Tobias Strohmann[1], Guillermo Requena[1,2], Eric Breitbarth[1]

[1] German Aerospace Center (DLR), Institute of Materials Research, Linder Hoehe, 51147, Cologne, Germany
[2] Metallic Structures and Materials Systems for Aerospace Engineering, RWTH Aachen University, 52062 Aachen, Germany

1 Supplementary Figure

As mentioned in the discussion of the paper, models focusing attention on the crack path rather than the crack tip field often erroneously detect crack tips in cases where the tip actually lies outside of the image. Figure S1 shows an example of this behavior. The model's field of view is restricted to the black box $0 \leq x \leq 40, -20 \leq y \leq 20$. The crack tip is located outside of the box at $x \approx 45$. The crack path focusing model *U-Net-2* makes a false crack tip prediction, whereas the crack tip field focusing model *ParallelNets-1* correctly detects no crack tip.

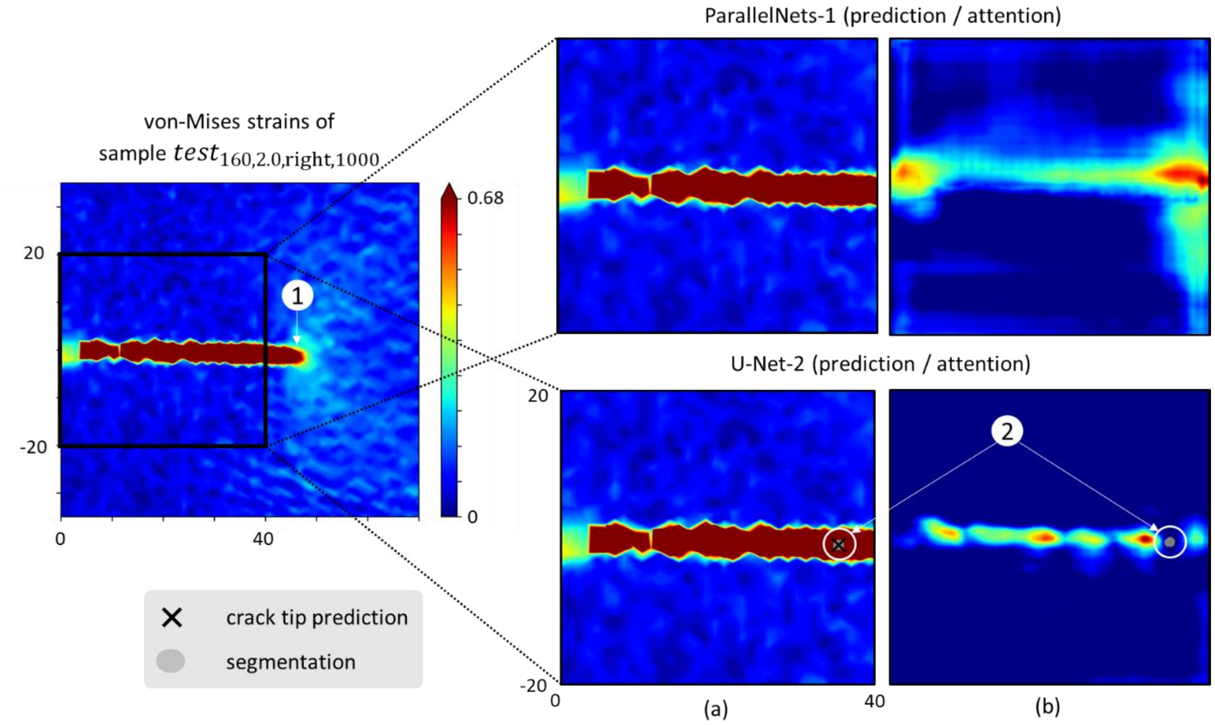

Figure S1: Erroneous crack tip segmentation. The crack tip is located outside the model's field of view (1). Column (a) shows the crack tip predictions of ParallelNets (top) and U-Net-2 (bottom). Column (b) shows the network attention of both models. ParallelNets-1 correctly detects no crack tip, whereas U-Net-2 erroneously detects a crack tip on the crack path (2).

2 Supplementary Videos

The evolution of the attention heatmaps obtained by the Grad-CAM method for the three neural networks ParallelNets-1, UNet-1, and UNet-2 and the three specimens can be seen in the supplementary videos together with the crack tip segmentation as the fatigue crack grows.

Video 1: Attention of ParallelNets-1 for the right-hand side of specimen $S_{160,2.0}$. Areas of high network attention are red and the crack tip segmentation is depicted in gray.

Video 2: Attention of ParallelNets-1 for the right-hand side of specimen $S_{160,4.7}$. Areas of high network attention are red and the crack tip segmentation is depicted in gray.

Video 3: Attention of ParallelNets-1 for the right-hand side of specimen $S_{950,1.6}$. Areas of high network attention are red and the crack tip segmentation is depicted in gray.

Video 4: Attention of UNet-1 for the right-hand side of specimen $S_{160,2.0}$. Areas of high network attention are red and the crack tip segmentation is depicted in gray.

Video 5: Attention of UNet-1 for the right-hand side of specimen $S_{160,4.7}$. Areas of high network attention are red and the crack tip segmentation is depicted in gray.

Video 6: Attention of UNet-1 for the right-hand side of specimen $S_{950,1.6}$. Areas of high network attention are red and the crack tip segmentation is depicted in gray.

Video 7: Attention of UNet-2 for the right-hand side of specimen $S_{160,2.0}$. Areas of high network attention are red and the crack tip segmentation is depicted in gray.

Video 8: Attention of UNet-2 for the right-hand side of specimen $S_{160,4.7}$. Areas of high network attention are red and the crack tip segmentation is depicted in gray.

Video 9: Attention of UNet-2 for the right-hand side of specimen $S_{950,1.6}$. Areas of high network attention are red and the crack tip segmentation is depicted in gray.
